# False Positivity of Non-Targeted Infections in Malaria Rapid Diagnostic Tests: The Case of Human African Trypanosomiasis

**DOI:** 10.1371/journal.pntd.0002180

**Published:** 2013-04-25

**Authors:** Philippe Gillet, Dieudonné Mumba Ngoyi, Albert Lukuka, Viktor Kande, Benjamin Atua, Johan van Griensven, Jean-Jacques Muyembe, Jan Jacobs, Veerle Lejon

**Affiliations:** 1 Department of Clinical Sciences, Institute of Tropical Medicine, Antwerp, Belgium; 2 Institut National de Recherche Biomédicale, Kinshasa, Democratic Republic of the Congo; 3 Programme National de Lutte contre la Trypanosomiase Humaine Africaine (PNLTHA), Kinshasa, Democratic Republic of the Congo; 4 Programme National de Lutte contre le Paludisme (PNLP), Kinshasa, Democratic Republic of the Congo; 5 Department of Biomedical Sciences, Institute of Tropical Medicine, Antwerp, Belgium; 6 Institut de Recherche pour le Développement, UMR 177 IRD-CIRAD INTERTRYP, Campus International de Baillarguet, Montpellier, France; Institute of Tropical Medicine, Belgium

## Abstract

**Background:**

In endemic settings, diagnosis of malaria increasingly relies on the use of rapid diagnostic tests (RDTs). False positivity of such RDTs is poorly documented, although it is especially relevant in those infections that resemble malaria, such as human African trypanosomiasis (HAT). We therefore examined specificity of malaria RDT products among patients infected with *Trypanosoma brucei gambiense*.

**Methodology/Principal Findings:**

Blood samples of 117 HAT patients and 117 matched non-HAT controls were prospectively collected in the Democratic Republic of the Congo. Reference malaria diagnosis was based on real-time PCR. Ten commonly used malaria RDT products were assessed including three two-band and seven three-band products, targeting HRP-2, *Pf*-pLDH and/or pan-pLDH antigens. Rheumatoid factor was determined in PCR negative subjects. Specificity of the 10 malaria RDT products varied between 79.5 and 100% in HAT-negative controls and between 11.3 and 98.8% in HAT patients. For seven RDT products, specificity was significantly lower in HAT patients compared to controls. False positive reactions in HAT were mainly observed for pan-pLDH test lines (specificities between 13.8 and 97.5%), but also occurred frequently for the HRP-2 test line (specificities between 67.9 and 98.8%). The *Pf*-pLDH test line was not affected by false-positive lines in HAT patients (specificities between 97.5 and 100%). False positivity was not associated to rheumatoid factor, detected in 7.6% of controls and 1.2% of HAT patients.

**Conclusions/Significance:**

Specificity of some malaria RDT products in HAT was surprisingly low, and constitutes a risk for misdiagnosis of a fatal but treatable infection. Our results show the importance to assess RDT specificity in non-targeted infections when evaluating diagnostic tests.

## Author Summary

Rapid diagnostic tests (RDT) for malaria are currently rolled-out as the backbone of parasite-based diagnosis, and their diagnostic accuracy is sufficiently high to substitute microscopy. One decade ago, attention has been given to occurrence of limited false positivity in a number of malaria RDTs, but false positivity of RDTs has remained poorly documented since then. In the last years, the number of available RDT products has dramatically increased and test performance has improved. False positivity may therefore not be perceived as a problem anymore. In this manuscript, we demonstrate that specificities of malaria rapid diagnostic tests detecting parasite antigens are seriously affected by human African trypanosomiasis (sleeping sickness), with values down to 11%. Malaria constitutes the main differential diagnosis of human African trypanosomiasis, and the false-positive results for malaria RDTs increase the risk of misdiagnosis or delayed diagnosis of human African trypanosomiasis which is a fatal but treatable infection.

## Introduction

Traditional diagnosis of malaria relies on microscopic detection of *Plasmodium* in thick blood films, which is labour-intensive, time-consuming and requires technical skills. Malaria rapid diagnostic tests (RDT) offer an attractive alternative for microscopy. They detect parasite antigens in blood through an antibody-antigen reaction, made visible by a red line on a nitrocellulose strip. Test formats include two- and three-band tests. Two band RDT products consist of a control line and a test line, three-band tests have a control line and 2 test lines, of which at least one usually targets a *Plasmodium falciparum* (*Pf*) specific antigen. The main target antigens are histidine rich protein 2 (HRP-2) specific for *Pf* and *Plasmodium* lactate dehydrogenase (pLDH), either detecting all human infective species (pan-pLDH) or specific for *Pf* (*Pf*-pLDH). Accuracies of RDTs are now such that they can substitute microscopy: average sensitivity and specificity have been estimated 95% for HRP-2 test lines, and respectively 93 and 99% for pLDH detection tests [1]. Of note, the HRP-2 antigen can persist for up to ≥6 weeks after (effectively treated) *Pf* infection [2], [3]. Besides this, false positivity has been attributed to the rheumatoid-factor [4]–[6], but has also been observed for anti-nuclear antibody, anti-mouse antibody and rapid plasma reagin positive samples [7], and has been attributed mainly to the HRP-2 test line. Limited false positivity has been noted in hepatitis C, schistosomiasis, toxoplasmosis, dengue, leishmaniasis and Chagas disease [7]–[9].

Occurrence of false positivity in RDTs may be particularly relevant for infections prevailing in malaria endemic regions, for which the differential diagnosis includes malaria. An example of such an infection is human African trypanosomiasis (HAT) [10]. Human African trypanosomiasis (HAT), or sleeping sickness, is a fatal but treatable infection caused by the parasites *Trypanosoma brucei* (*T.b*.) *gambiense* and *rhodesiense*, which are transmitted by tsetse flies. Association of HAT with a strong polyclonal B-cell activation and with rheumatoid factor-like anti-immunoglobulin antibodies has been reported [11]. Furthermore, *T.b. gambiense* infection has been shown to decrease the specificity of antibody detection tests for HIV diagnosis [12]. Based on these reports we put forward that HAT may be associated with false positivity in malaria RDTs. The objective of this study was therefore to examine the specificity of some commonly used malaria RDT products in HAT.

## Materials and Methods

### Ethics statement

Before enrolment into the study, participants gave written informed consent. Parents or guardians provided consent on behalf of all child participants. Ethical clearance for the study was obtained from the institutional review board of ITM and the ethical committees of the University Hospital in Antwerp, Belgium (study registration number B30020108363) and of the Ministry of Health of the Democratic Republic of the Congo (DR Congo).

### Study population and specimen collection

HAT patients and paired non-HAT endemic controls were prospectively included in DR Congo, Bandundu Province between July and December 2010. Malaria transmission in most parts of DR Congo, including in Bandundu Province, is high (>1 case per 1000 population) and DR Congo is considered as suffering from the highest malaria burden in Africa [13], [14]. Moreover, almost 80% of all notified HAT cases originate from DR Congo, and almost half of them are detected in Bandundu where the HAT prevalence is estimated at 0.36% [15]. Participants were identified during HAT screening activities of the dedicated HAT mobile team of Masi-Manimba, or included at the HAT treatment centres of Masi-Manimba and Bonga-Yasa. Inclusion criteria for HAT patients were the presence of trypanosomes in blood, lymph and/or cerebrospinal fluid (irrespective of disease stage), and being 12 years or older. Exclusion criteria were pregnancy, being previously treated for HAT and being moribund. To each HAT patient, a control was matched, fulfilling the following criteria: same gender and age and being permanent resident from the same or a neighbouring village. Inclusion criteria for controls were absence of clinical evidence for HAT (no swollen lymph nodes or neurological symptoms), absence of trypanosome specific antibodies in whole blood detected by card agglutination test for trypanosomiasis [16]; no trypanosomes in blood detected by the mini anion exchange centrifugation technique [17] and being 12 years or older. Exclusion criteria were identical as for HAT patients. From the participants, clinical and epidemiological parameters in conjunction to HAT were collected, including the use of anti-malaria medication in the month prior to inclusion. Blood was drawn on EDTA and on heparin. For conservation of DNA, 0.5 ml of EDTA blood was mixed with an equal volume of GE buffer (6 M guanidium, 0.2 M EDTA, pH 8.0) and stored at ambient temperature until DNA extraction. Aliquots of blood taken on EDTA and of plasma prepared from the blood taken on heparin were snap frozen in liquid nitrogen and shipped to ITM where specimens were stored at −70°C until use. Two thick and thin blood films were prepared and Giemsa stained; one set remained in Kinshasa, the double was shipped to ITM.

### Reference tests

DNA was extracted from EDTA blood mixed with GE buffer using the Maxwell 16 DNA Purification Kit (Promega, Madison, Wisconsin, USA). A four primer real-time PCR was performed for detecting single and mixed infections of *Plasmodium falciparum* (*Pf*), *Plasmodium vivax* (*Pv*), *Plasmodium malariae* (*Pm*) and *Plasmodium ovale* (*Po*) as previously described [18], [19]. A negative and positive control for each *Plasmodium* species was included for each test run. If *Pf*-PCR, *Pv*-PCR, *Pm*-PCR or *Po*-PCR was positive, malaria-PCR was considered positive, if negative for all species, malaria-PCR was considered negative. Standard microscopy was performed on thick blood films by an expert microscopist. Parasite density was assessed by counting asexual parasites against 200 white blood cells (WBC) in thick blood films and converted into parasites/µl using the standard of 8000 WBC/µl [20].

### Index tests

In total, ten rapid diagnostic tests for malaria were evaluated. Malaria RDTs in a cassette format were selected based on (i) demonstrated diagnostic accuracy [7], [21] and/or (ii) use by the national malaria control programme and non-governmental organizations in trypanosomiasis endemic countries. In addition, RDTs detecting *Pf*-pLDH were added. The following two-band tests were used: 1° Paracheck Pf (Orchid Biomedical Systems, Goa, India); 2° ICT Malaria Pf Cassette Test (ICT Diagnostics, Cape Town, Republic of South Africa); 3° Advantage Pan Malaria Card (J. Mitra & Co Pvt. Ltd., New Delhi, India). The three-band tests were: 4° Malaria Antigen Pf (HRP-2/pLDH) (Standard Diagnostics Inc., Hagal-Dong, Korea); 5° SD malaria Ag Pf/Pan (Standard Diagnostics Inc); 6° SD Malaria Antigen Pf (Standard Diagnostics Inc.); 7° ICT Malaria Combo Cassette Test (ICT Diagnostics); 8° Carestart Malaria HRP2/pLDH (Pf/Pan) Combo Test (Acces Bio, New Jersey, USA); 9° Carestart Malaria pLDH (Pf/pan) (Acces Bio); 10° First Response Malaria Ag (pLDH/HRP2) Combo Rapid Diagnostic Test (Premier Medical Corporation Ltd., Daman, India). Tests were performed according to the instructions of the manufacturers, using thawed EDTA blood. Three experienced readers who were blinded to each other's and to other results, except for the HAT status, read the RDT results, at daylight and within the prescribed delay. After reading, RDT results were immediately photographed. Test line intensities were scored as negative (no line visible), faint (barely visible), weak (paler than the control line), medium (equal to the control line), strong (stronger than the control line). Invalid tests (no control line) were repeated. Based on the three scores, a consensus line intensity score was established. If two out of three readers had scored the line intensity the same, this was taken as the consensus. If the three readers had scored the intensity differently, the photograph was used for taking the final decision. For analysis of test line results, faint, weak, medium and strong line intensities were considered positive, absence of test lines was recorded as negative. Interpretation of the RDT result was as prescribed by the manufacturer. An RDT was considered negative for malaria when all test lines were negative, and positive when at least 1 test line was positive.

### Rheumatoid factor

The presence and concentration of rheumatoid factor in plasma of all *Pf*-PCR negatives was measured using the VITROS chemistry Products RF reagent on the VITROS 5600 Integrated system (Ortho-Clinical Diagnostics, Buckinhamshire, UK). The procedure consists of an antigen-antibody reaction occurring between rheumatoid factor from the test sample and denatured human IgG adsorbed to latex particles from the test reagent, resulting in agglutination. The agglutination is detected as an absorbance change, with the magnitude of the change being proportional to the quantity of rheumatoid factor. The detection limit was 9 IU/ml, as suggested in the instructions, values ≥12 IU/ml were considered abnormal.

### Result analysis

For calculation of specificity and sensitivity, PCR was used as the reference method (Supporting information file S1). Specificity of HRP-2 or *Pf*-pLDH lines was assessed respectively as the number of HRP-2 or *Pf*-pLDH line negatives/number of *Pf*-PCR negatives; sensitivity was defined as the numbers of HRP-2 or *Pf*-pLDH line positives/number of *Pf*-PCR positives. Likewise, specificity of pan-pLDH lines was calculated as the number of pan-pLDH negatives/number of malaria PCR negatives and the sensitivity as the number of pan-pLDH positives/number of malaria PCR positives. Specificity of three-band RDTs for diagnosis of malaria was calculated as the number of RDT negatives/number of malaria PCR negatives or number of RDT negatives/number of *Pf*-PCR negatives if the RDT consisted only *Pf* specific test lines. Proportions were calculated with 95% exact confidence intervals (CI). In a primary analysis, differences between proportions were tested for significance using the Fisher exact probability test (STATA 10.0), in order to avoid data loss and as the effect of matching, which was done for another study component, was expected to be small on RDT performance. For comparison of specificity, this primary analysis was followed by a secondary analysis using McNemar chi square, after matching controls and HAT again taking into account the PCR result. For sensitivity, the low number of PCR-matched samples precluded meaningful comparison with the McNemar test. Inter-reader reliabilities were assessed for the test results expressed as positive and negative line readings and kappa values for each pair of readers were calculated.

## Results

### Study population and reference tests

An overview of the study population and reference test results is shown in table 1. In total, 117 HAT patients and 117 controls were included in the study. Median age was 28 years and 45% of the study participants were male. Significantly more HAT patients than controls had taken anti-malaria drugs in the month prior to inclusion (*p* = 0.01). Twenty-two (9.9%, CI 5.9–13.8) thick blood films were positive for *Plasmodium*. Due to low *Plasmodium* parasite densities, the *Plasmodium* species could be defined only in 16 thin blood films, and was *Pf* in 15, *Po* in one. In total, 79/233 participants were malaria-PCR positive (1/234 sample of blood on GE buffer missing), including all 22 that were thick blood film positive. Species identification as defined by PCR were 87% *Pf* (69/79), followed by *Po* (26.6%, 21/79), *Pm* (21.5%, 17/79) and *Pv* (1.3%, 1/79).

**Table 1:** Study population and reference test results.

	Control		HAT		*p*-value
	n/N		n/N		
% male	53/117	45%	53/117	45%	
Median age (IQR)	117	28 (19–41)	117	28 (20–42)	
Anti-malaria drugs (CI)	20/116	17.2% (10.3–24.2)	36/112	32.1% (23.3–40.9)	0.01
Thick blood film positive	13/112	11.6% (5.6–17.6)	9/111	8.1% (3.0–13.3)	0.5
Median *Plasmodium* density/µl in positive thick blood film (IQR)	10/13	59 (15–143)	6/9	109 (52–296)	
Malaria PCR positive	44/117	37.6% (28.7–46.5)	35/116	30.2% (21.7–38.7)	0.4
*Pf*-PCR positive	38/117	32.5% (23.9–41.1)	31/116	26.7% (18.6–34.9)	0.3

IQR: interquartile range, CI: confidence interval.

### Accuracy of *Pf*-pLDH test lines for diagnosis of *P. falciparum*

**Figure 1:**
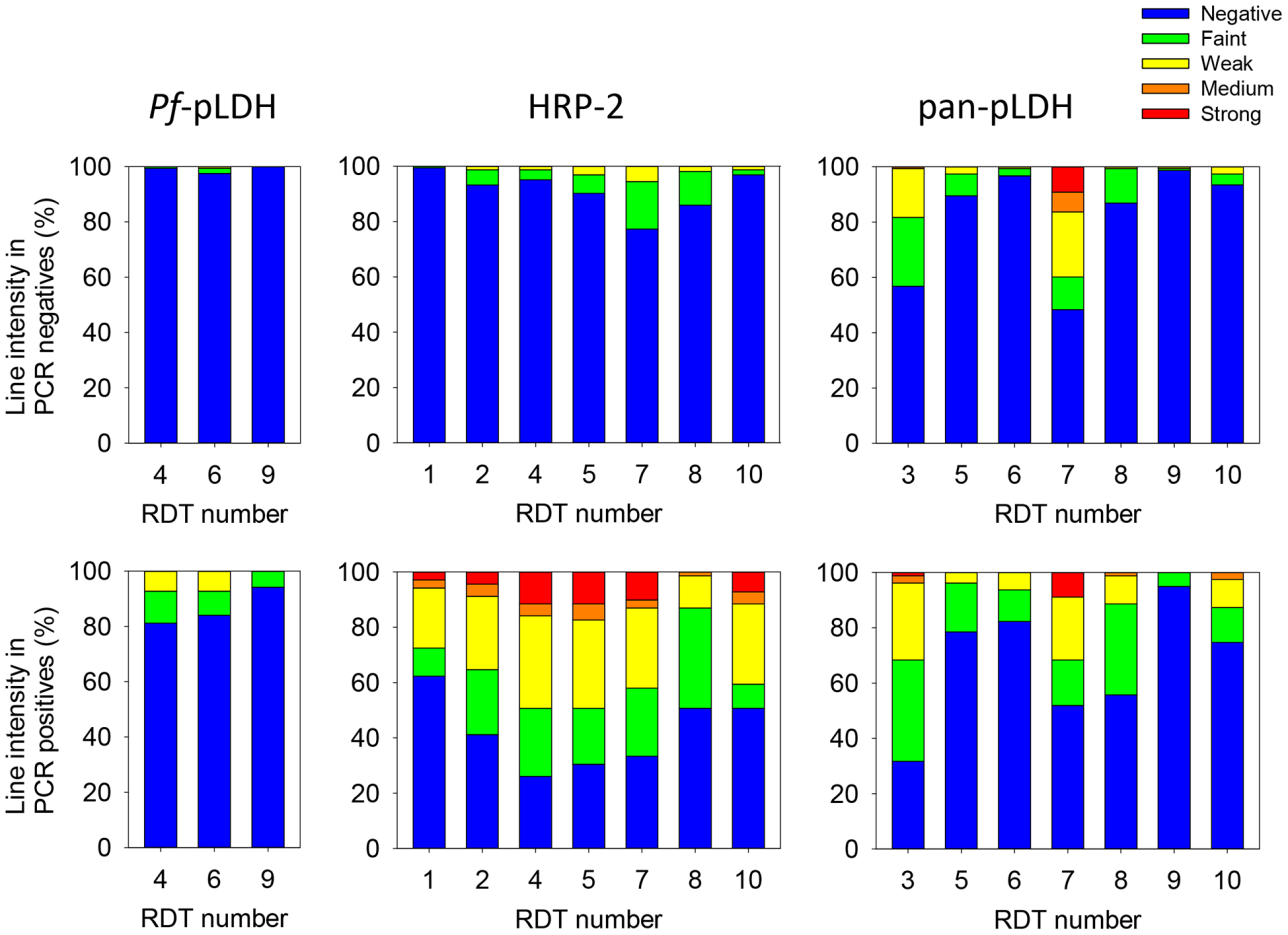
Line intensities for Pf-pLDH, HRP-2 and pan-pLDH in PCR negative and positive study participants. Pf-pLDH: left, HRP-2: middle, pan-pLDH: right. PCR negative: upper panels, PCR positive: lower panels. RDT number: 1° Paracheck Pf; 2° ICT Malaria Pf Cassette Test; 3° Advantage Pan Malaria Card; 4° Malaria Antigen Pf (HRP-2/pLDH); 5° SD malaria Ag Pf/Pan; 6° SD Malaria Antigen Pf; 7° ICT Malaria Combo Cassette Test; 8° Carestart Malaria HRP2/pLDH (Pf/Pan) Combo Test; 9° Carestart Malaria pLDH (Pf/pan); 10° First Response Malaria Ag (pLDH/HRP2) Combo Rapid Diagnostic Test.

Specificity of *Pf*-pLDH test lines in three malaria RDT products was 97.5–100% (table 2). There was no significant difference in specificity between controls and HAT patients. False positive test lines were faint (1–3/163) or weak (1/163, figure 1). Sensitivities of *Pf*-pLDH test were respectively 21.1–28.9% in controls (n = 38) and 9.7% in HAT patients (n = 31). True positive reactions consisted of faint (4–8/69) and weak lines (0–5/69, figure 1). For none of the RDT products, a significant difference in sensitivity between controls and HAT patients was observed. On the complete series of samples tested (n = 233), there was almost perfect agreement between at least two out of three readers assessing the *Pf*-pLDH lines (maximal kappa values between two readers of 0.89–0.96).

**Table 2:** RDT test line specificities for diagnosis of malaria infection in controls and HAT.

	Malaria RDT	Test line	% specificity controls^a^	% specificity HAT^b^	*p-*value^c^
1	Paracheck Pf	HRP-2	100 (95.4–100)	98.8 (93.5–100)	1
2	ICT Malaria Pf Cassette Test	HRP-2	98.7 (93.1–100)	87.9 (80.0–94.1)^d^	0.009
3	Advantage Pan Malaria Card	pan-pLDH	86.3 (76.2–93.2)	30.0 (20.3–41.3)	<0.001
4	Malaria Antigen Pf (HRP-2/pLDH)	HRP-2	97.5 (91.1–99.7)	83.3 (73.6–90.6)	0.003
		*Pf*-pLDH	100 (95.4–100)	98.8 (93.5–100)	1
		Combined	97.5 (91.1–99.7)	83.3 (73.6–90.6)	0.003
5	SD malaria Ag Pf/Pan	HRP-2	100 (95.4–100)	90.5 (82.1–95.8)	0.007
		pan-pLDH	100 (95.1–100)	80.0 (69.6–88.1)	<0.001
		Combined	100 (95.1–100)	76.3 (65.4–85.1)	<0.001
6	SD Malaria Antigen Pf	*Pf*-pLDH	97.5 (91.2–99.7)	97.6 (91.6–99.7)	1
		pan-pLDH	97.3 (90.5–99.7)	96.3 (89.4–99.2)	1
		Combined	97.3 (90.5–99.7)	96.3 (89.4–99.2)	1
7	ICT Malaria Combo Cassette Test	HRP-2	87.3 (78.0–93.8)	67.9 (56.8–77.6)	0.005
		pan-pLDH	86.3 (76.2–93.2)	13.8 (7.1–23.3)	<0.001
		Combined	79.5 (68.4–88.0)	11.3 (5.3–20.3)	<0.001
8	Carestart Malaria HRP2/pLDH (Pf/Pan) Combo Test	HRP-2	92.6 (84.2–97.2)	79.8 (69.6–87.7)	0.03
		pan-pLDH	98.6 (92.6–99.8)	76.3 (65.4–85.1)	<0.001
		Combined	91.8 (83.0–96.9)	61.3 (50.5–72.0)	<0.001
9	Carestart Malaria pLDH (Pf/pan)	*Pf*-pLDH	100 (95.4–100)	100% (95.7–100)	1
		pan-pLDH	100 (95.1–100)	97.5 (91.3–99.7)	0.5
		Combined	100 (95.1–100)	97.5 (91.3–99.7)	0.5
10	First Response Malaria Ag (pLDH/HRP2) Combo Rapid Diagnostic Test	HRP-2	98.7 (93.1–100)	95.2 (88.3–98.7)	0.4
		pan-pLDH	98.6 (92.6–99.8)	88.8 (79.7–94.7)	0.02
		Combined	97.3 (90.5–99.7)	86.3 (76.7–92.9)	0.02

a HRP-2 and Pf-pLDH N = 79 *Pf*-PCR negative controls, pan-pLDH test lines n = 73 malaria-PCR negative controls.

b HRP-2 and *Pf*-pLDH N = 84/85 *Pf*-PCR negative HAT patients (1 blood sample missing), pan-pLDH N = 80/81 malaria-PCR negative HAT patients (1 blood sample missing).

c *p* values for Fisher exact test.

d N = 83. The test line for 1 sample could not be scored due to poor background clearance of the test strip.

### Accuracy of HRP-2 test lines for diagnosis of *P. falciparum*

Specificity of HRP-2 test lines in seven malaria RDT products for controls and HAT patients are summarized in table 2. In controls, HRP-2 test line specificity was between 87.3 and 100% while in HAT, it was between 67.9 and 98.8%. Significantly lower specificity of the HRP-2 test line in HAT compared to controls was observed for five out of seven RDT products. After matching, the difference in specificity was lost for the Carestart Malaria HRP2/pLDH (Pf/Pan) Combo Test. In participants not having taken anti-malaria drugs prior to inclusion, specificities in controls (n = 63) and HAT (n = 53) ranged respectively 88.5–100% and 67.9–100%, and specificity of HRP-2 test lines remained significantly lower in HAT compared to negative controls for two RDT products (ICT Malaria Pf Cassette Test, ICT Malaria Combo Cassette Test). In Malaria Antigen Pf (HRP-2/pLDH), SD malaria Ag *Pf*/pan and Carestart Malaria HRP2/pLDH (Pf/Pan) Combo Test, a similar tendency towards lower specificity of HRP-2 in HAT was observed, but the difference lost significance. Line intensity scores are shown in figure 1. False positivity was mainly due to presence of faint HRP-2 test lines (1–28/163) and to a lesser extent due to weak HRP-2 test lines (0–9/163). No medium or strong false positive HRP-2 test lines were observed. With respectively 22/163 samples being HRP-2 false positive in ≥two RDT products and 13/163 in ≥three RDT products, false positivity appeared to be at random distributed over the samples.

Sensitivity of HRP-2 test lines in *Pf*-PCR positive controls (n = 38) and HAT patients (n = 31) ranged between 47.4–71.1% and 25.8–74.2% respectively. No significant difference in sensitivity of HRP-2 was observed between controls and HAT patients. The majority of positive lines scored weak (8–23/69), medium (1–4/69) or strong (0–8/69, Figure 1), with the exception of the Carestart Malaria HRP2/pLDH (Pf/Pan) Combo Test where mainly faint test lines (25/69) were seen (Figure 1).

On the complete series of samples tested (n = 233), there was substantial to almost perfect agreement between at least two readers in HRP-2 line intensity (maximal kappa value between two readers of the seven RDTs were 0.75–0.95).

### Accuracy of pan-pLDH test lines for diagnosis of *Plasmodium*

For pan-pLDH tests as well, a significant lower specificity in HAT patients compared to controls was observed with five out of seven RDT products (Table 2). These differences were confirmed after taking into account matching. Pan-pLDH test line specificities in controls ranged between 86.3–100% versus between 13.8–97.5% in HAT patients. False positive pan-pLDH test lines were mainly of faint or weak line intensity (respectively 1–38 and 1–36/153, figure 1), but false positive medium (1–11/153) and strong (14/153) intensity pan-pLDH test lines were observed as well, especially with ICT Malaria Combo Cassette Test. With respectively 62/153 samples being pan-pLDH false positive in ≥two RDTs and 29/153 in ≥three RDTs, it appeared that the same samples were responsible for most false positives in the different RDTs.

Sensitivity of pan-pLDH test lines in malaria-PCR positive controls (n = 44) was 0–63.6%, in HAT patients (n = 35) it was 11.4–77.1%. Sensitivity in controls was significantly lower than in HAT patients for two out of seven RDTs: ICT Malaria Combo Cassette Test (respectively 25.0% (13.2–40.3) versus 77.1% (58.9–89.6), *p*<0.001) and Carestart Malaria pLDH (Pf/pan) (respectively 0.0% (0–8.0) versus 11.4% (3.2–26.7), *p*<0.04). The majority of positive line intensities scored only faint (4–29/79) or weak (0–22/79), but some medium (0–2/79) or strong tests line intensities (0–7/79) were observed as well. On all 233 samples tested, there was substantial to almost perfect agreement between at least two readers (maximal kappa values between 2 readers of the 7 RDTs of 0.79–1).

### Specificity of the combined test result in three-band RDTs

Specificity of the combined test result in the seven three-band RDTs in controls and HAT patients are summarized in table 2. For five out of seven RDT products, combining HRP-2 with either *Pf*-pLDH or pan-pLDH, specificity was significantly lower in HAT patients compared to controls. These differences were confirmed after taking into account matching. In both tests combining *Pf*-pLDH with pan-pLDH test lines (SD Malaria Antigen and Carestart Malaria pLDH (Pf/pan)), no difference between HAT patients and controls was observed, and specificities were 96.3 to 100%.

After subtraction of patients who were on anti-malaria drugs prior to sampling, specificities in controls (n = 55) and HAT (n = 50) for the different RDTs ranged between 78.2–100% and 12.0–100% respectively, and remained significantly lower in HAT patients compared to controls for three out of five three-band RDT products incorporating HRP-2 test lines (SD Malaria Antigen, ICT Malaria Combo Cassette Test, Carestart Malaria HRP2/pLDH (Pf/Pan) Combo Test). In Malaria Antigen Pf (HRP-2/pLDH) and First Response Malaria Ag (pLDH/HRP2) Combo Rapid Diagnostic Test, a similar tendency towards lower specificity in HAT was observed, but the difference lost significance.

### Rheumatoid factor

Proportions of rheumatoid factor positive samples were 7.6% (6/79) among controls (maximum concentration 47 IU/ml) and 1.2% among HAT patients (1/84, maximum 45 IU/ml) respectively. This difference was not statistically different (*p* = 0.06). There was no association between false positivity of any of the HRP-2, pan-pLDH, or *Pf*-pLDH test lines and the presence and concentration of rheumatoid factor (*p* values ranging 0.3 to 1).

## Discussion

We demonstrated that specificity of seven out of ten malaria RDT products was significantly lower in HAT patients compared to controls. RDT products generated false positive test results in various proportions. The problem was most pronounced with pan-pLDH test lines and occurred to a lesser extent with HRP-2 test lines. The *Pf*-pLDH test lines were not affected. Rheumatoid factor was not associated with false positivity.

Our study has some limitations. It was set up to test the specificity of the RDTs and not their sensitivity to detect clinical malaria. Furthermore, our data only apply to chronic *T.b. gambiense* HAT, from which we collected specimens in a single HAT focus, and not to the more acute disease form caused by *T.b. rhodesiense*, and did not address other diseases that might influence malaria RDT specificity. Further, to assess the influence of the persistence of HRP-2, we did *a posteriori* calculations for specificity excluding patients with history of use of anti-malaria medication one month prior to inclusion. However, given reported persistence of HRP-2 for up to 6 weeks or even longer, this one-month period may have been too short to rule out all interference by HRP-2 persistence [2], [3]. This exclusion was further based on self-reported treatment history but not on parasitologically demonstrated infection in the month prior to sampling. Although the effect on test specificities was limited, the reduced numbers in each group resulted in loss of significance for some tests. Finally, the exact nature of the confounding factor in HAT was not identified.

Despite these limitations, this is the first study carrying out an evaluation of specificity of malaria RDT products in HAT patients. Problems with specificity of malaria RDT products for other non-malarial infectious pathogens have been observed for dengue, schistosomiasis, leishmaniasis and Chagas disease but on limited sample numbers only [7]. Other strengths are the high number of HAT patients tested, and the fact that the study was organised prospectively. Furthermore, RDTs were run side-by- side in a reference setting, limiting sources of variation. PCR was preferred as reference test over microscopy due to its higher sensitivity to detect *Plasmodium* infection [19], enabling us to eliminate microscopy false negatives from the specificity calculations. Taking microscopy as a more conservative reference test gives a relative underestimate of the specificity of the RDTs (Supporting information file S2). Finally, matching of controls and HAT patients based on the PCR results again confirmed differences in specificity observed with the Fisher exact test, despite data loss.

Although the number of RDT products including *Pf*-pLDH test lines was limited to three, we confirm that the *Pf*-pLDH test line seems to be less prone to false positivity, but our findings are in contrast with the high specificity of the pan-pLDH test line previously described [6], [8], [9].

Previous reports have mainly focused on the presence of rheumatoid factor as a reason for false positive HRP-2 test line reactions [4]–[6]. Rheumatoid factor was present in 1.2% of HAT patients only, excluding it as the major cause of false positive malaria RDTs in this patient group. Our results contradict previous findings of rheumatoid factor-like anti-immunoglobulin antibodies in 74% of HAT patients [11]. It is not clear if differences in reagent and methodology, rabbit immunoglobulin in a solid phase anti globulin assay previously [22], versus human IgG in a latex agglutination assay in our measurements, account for these different results. It also seems unlikely that the high IgG concentrations, known to be present in *T.b. gambiense* HAT sera [23] and known to possibly interfere with quantitation of rheumatoid factor, would account completely for the absence of measurable rheumatoid factor. Although we presently did not assess anti-nuclear antibody, anti-mouse antibody or rapid plasma reagin to be co-responsible for false positivity [7], the abnormally high immunoglobulin concentrations in blood of HAT patients [23] may account on their own for the variability of false positive reactions of HAT specimens in malaria RDTs, to which some brands of tests may be more susceptible than others, depending on the reaction conditions and antibodies used for antigen detection.

Although it cannot be entirely ruled out, it seems improbable that problems of quality among RDT test lots would have accounted for the low malaria RDT specificity in HAT. All RDT products were stored according to the instructions of the manufacturers and were used before their expiry date. Lot to lot variation was assessed for ICT Malaria Combo Cassette Test and obtained specificities were similarly low for the second as for the first lot tested (data not shown). Cross-reactions of specific trypanosome antigens present in blood of HAT patients with the antibodies used in the malaria RDTs also seems unlikely, because of the large variation of proportions of false positive results between the different RDT products.

Although malaria constitutes the main differential diagnosis of HAT [10], this is the first study evaluating accuracy of malaria antigen detection RDTs in HAT. Although in areas with high malaria endemicity such as Bandundu, people of 12 years or older have acquired sufficient immunity against malaria parasites, so that if they have fever or feel unwell the reason should not be automatically ascribed to malaria even if the RDT, or any other diagnostic test, is positive, mistaking HAT for malaria is probably a frequent event. This is indirectly supported by our finding that HAT patients had taken significantly more anti-malaria treatment than controls. In DR Congo, RDTs for malaria are deployed since 2010, with Paracheck Pf being reported to be most often used in 2010 [24], and SD malaria Ag Pf/Pan being actually recommended by the national control program for malaria. Without doubt, other malaria RDTs circulate. Problems with specificity of some malaria RDTs may increase the risk of misdiagnosis or delayed diagnosis of HAT, which is already an important problem [25]. Our findings therefore show the interest of constituting a HAT specimen test panel for evaluation of false positive reactions and including HAT specimens in RDT product testing rounds [7]. This should not only be done for malaria, but also for HIV antibody detection RDTs tests, for which low specificity during HAT infection has been reported earlier [12]. Furthermore, in HAT endemic areas, awareness among health personnel of the possibility of HAT infection, even if tests for other diseases are positive, should be increased. Rapid tests for screening on HAT specific antibodies are now available, and allow identification of HAT suspects even at the level of the health centre [26].

We withhold from proposing a particular malaria RDT to be used in HAT and malaria co-endemic regions since actually we cannot present relevant data on sensitivity of the RDTs in clinical malaria. Parasite densities in immune patients exhibiting symptoms range from 2,500 in infants to 30,000/µl in adults [2]. The highest parasite densities observed in our participants were 978 and 694 parasites/µl, and corresponding blood samples tested positive in all malaria RDTs (data not shown). Although we confirm higher test sensitivity for HRP-2 compared to pLDH test lines [1], the low sensitivity of malaria RDTs in the actual samples can be ascribed to low parasite densities and is of less clinical importance for malaria immune patients. Sensitivity of malaria RDT therefore remains to be tested in a clinically relevant target population [27] before an appropriate malaria RDT for a HAT endemic area can be proposed.

Our results emphasize the importance, in general, to explore the impact of non-targeted infections when evaluating diagnostic tests.

## Supporting Information

File S1 Calculation of specificity and sensitivity using PCR as the reference method. HAT Human African trypanosomiasis; C control; *Pf Plasmodium falciparum*; Sens sensitivity; Spec specificity
(PPTX)

File S2 Specificities of RDTs for malaria diagnosis in controls and HAT using microscopy as a reference.
(DOC)
