# Supplementary material for: False Positivity of Non-Targeted Infections in Malaria Rapid Diagnostic Tests: The Case of Human African Trypanosomiasis
Source: PLoS Negl Trop Dis. 2013 Apr 25;7(4):e2180. doi: 10.1371/journal.pntd.0002180 (PMC3636101; doi:10.1371/journal.pntd.0002180)
Supplement: File S1 — Calculation of specificity and sensitivity using PCR as the reference method. HAT Human African trypanosomiasis; C control; Pf Plasmodium falciparum; Sens sensitivity; Spec specificity (PPTX) [file pntd.0002180.s001.pptx]

## Slide 1
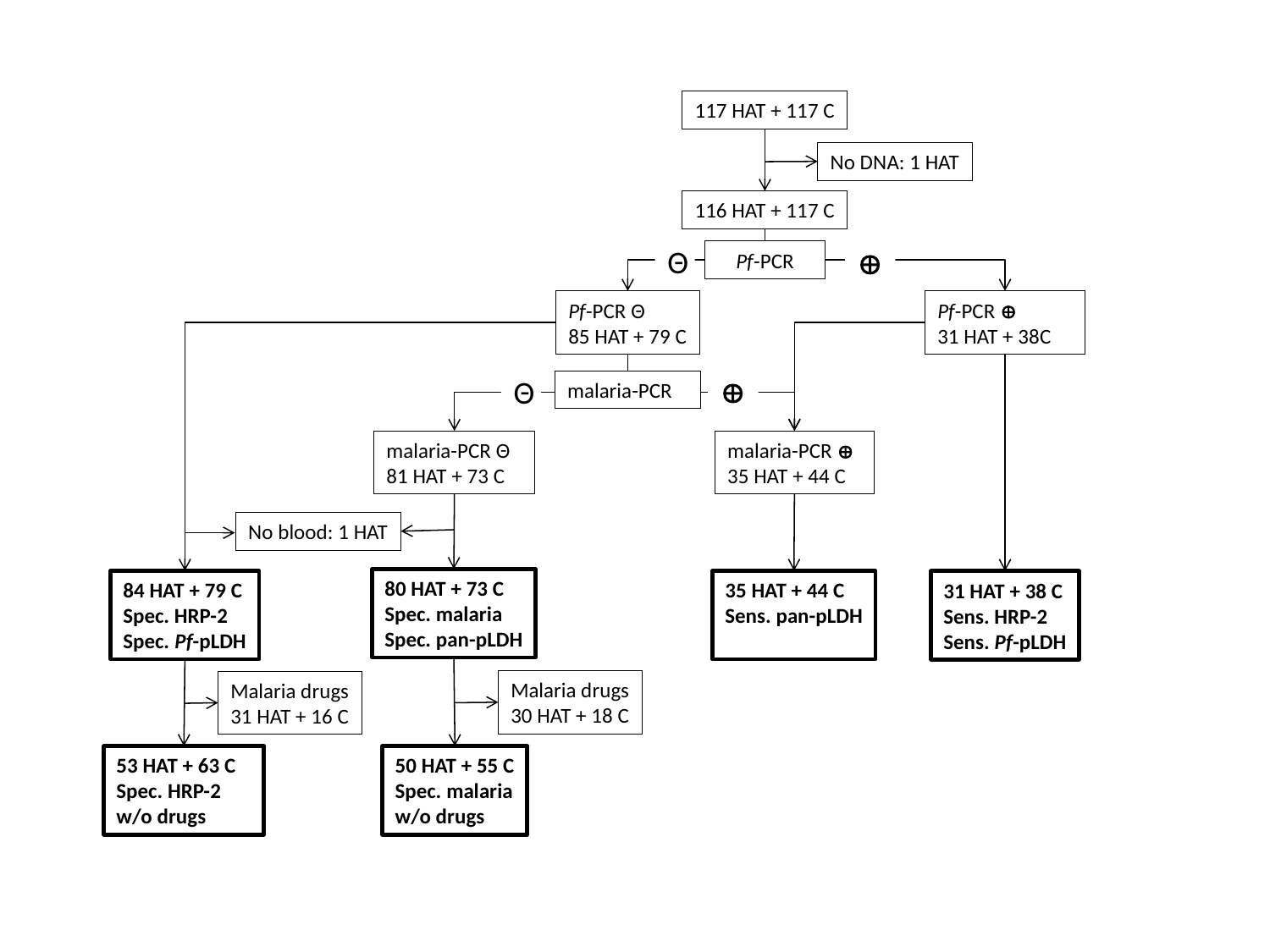

117 HAT + 117 C
No DNA: 1 HAT
116 HAT + 117 C
Θ

Pf-PCR
Pf-PCR Θ
85 HAT + 79 C
Pf-PCR 
31 HAT + 38C

Θ
malaria-PCR
malaria-PCR Θ
81 HAT + 73 C
malaria-PCR 
35 HAT + 44 C
No blood: 1 HAT
80 HAT + 73 C
Spec. malaria
Spec. pan-pLDH
84 HAT + 79 C
Spec. HRP-2
Spec. Pf-pLDH
35 HAT + 44 C
Sens. pan-pLDH
31 HAT + 38 C
Sens. HRP-2
Sens. Pf-pLDH
Malaria drugs
30 HAT + 18 C
Malaria drugs
31 HAT + 16 C
53 HAT + 63 C
Spec. HRP-2
w/o drugs
50 HAT + 55 C
Spec. malaria
w/o drugs
