# Supplementary material for: False Positivity of Non-Targeted Infections in Malaria Rapid Diagnostic Tests: The Case of Human African Trypanosomiasis
Source: PLoS Negl Trop Dis. 2013 Apr 25;7(4):e2180. doi: 10.1371/journal.pntd.0002180 (PMC3636101; doi:10.1371/journal.pntd.0002180)
Supplement: File S2 — Specificities of RDTs for malaria diagnosis in controls and HAT using microscopy as a reference. (DOC) [file pntd.0002180.s002.doc]

|  | Malaria RDT | Control n=99 | HAT n=101^a^ | *p-value***^b^** |
| --- | --- | --- | --- | --- |
| 1 | Paracheck Pf | 89.9 (82.2-95.0) | 96.0 (90.2-98.9) | 0.1 |
| 2 | ICT Malaria Pf Cassette Test | 85.9 (77.4-92.0) | 78.8 (69.4-86.4)^c^ | 0.3 |
| 3 | Advantage Pan Malaria Card | 72.7 (62.9-81.2) | 29.7 (21.0-39.6) | <0.001 |
| 4 | Malaria Antigen Pf (HRP-2/pLDH) | 81.8 (72.8-88.9) | 73.3 (63.5-81.6) | 0.2 |
| 5 | SD malaria Ag Pf/Pan | 82.8 (73.9-89.7) | 65.3 (55.2-74.5) | 0.006 |
| 6 | SD Malaria Antigen Pf | 96.0 (90.0-98.9) | 95.0 (88.8-98.4) | 1 |
| 7 | ICT Malaria Combo Cassette Test | 70.7 (60.7-79.4) | 11.9 (6.3-19.8) | <0.001 |
| 8 | Carestart Malaria HRP2/pLDH (Pf/Pan) Combo Test | 79.8 (70.5-87.2) | 52.5 (42.3-62.5) | <0.001 |
| 9 | Carestart Malaria pLDH (Pf/pan) | 96.0 (90.0-98.9) | 97.0 (91.6-99.4) | 0.7 |
| 10 | First Response Malaria Ag (pLDH /HRP2) Combo Rapid Diagnostic Test | 86.9 (78.6-92.8) | 80.2 (71.1-87.5) | 0.3 |

^a^ Specificity of RDTs was evaluated in 101/102 thick blood film negative samples since 1 blood sample was missing.

**^b^** *p* values for Fisher exact test

^c^ N=99. The test line for 2 samples could not be scored due to poor background clearance of the test strip.
